# Evaluation of An I226R and A137R deletion mutant from a genotype I/II recombinant African swine fever virus strain as a live attenuated vaccine in pigs

**DOI:** 10.1186/s13567-026-01827-6

**Published:** 2026-08-28

**Authors:** Han Zhang, Qixuan Li, Nannan Li, Ying Wang, Tianying Hou, Hongliang Wang, Rongliang Hu, Yanyan Zhang, Shoufeng Zhang, Faming Miao

**Affiliations:** 1 State Key Laboratory of Pathogen and Biosecurity, Key Laboratory of Prevention & Control for African Swine Fever and Other Major Pig Diseases, Ministry of Agriculture and Rural Affairs, Academy of Military Medical Sciences, Changchun, China; 2 Institute of Animal Husbandry and Veterinary Medicine, Heilongjiang Academy of Agricultural Reclamation Sciences, Harbin, China

**Keywords:** African swine fever virus, gene deletion, *I226R*, *A137R*, recombinant strain

## Abstract

The emergence of highly lethal genotype I/II recombinant African swine fever virus (ASFV) strains in China has rendered existing genotype II-based live attenuated vaccines ineffective, underscoring the urgent need for novel vaccine candidates. Although single-gene deletions of *A137R* or *I226R* have shown promise against genotype II strains, their efficacy and safety in the context of recombinant strains remain unexplored. Using homologous recombination, we constructed a single gene-deleted mutant (JX23-02ΔI226R) and a double gene-deleted mutant (JX23-02ΔI226RΔA137R) from the genotype I/II recombinant ASFV strain JX23-02. The replication kinetics, pathogenicity, immunogenicity, and protective efficacy of these mutants were evaluated in vitro and in pigs. Both deletion mutants exhibited significantly reduced replication in porcine alveolar macrophages. Immunization with JX23-02ΔI226R resulted in 40% survival, and surviving pigs were protected against lethal challenge. In contrast, JX23-02ΔI226RΔA137R was fully attenuated: all immunized pigs survived challenge without any clinical signs (maximum rectal temperature 40.2 °C) and developed detectable p54-specific antibody responses. Moreover, viral shedding and tissue viral loads were markedly lower in the double-deletion group than in the single-deletion group. The double gene-deleted mutant JX23-02ΔI226RΔA137R represents a promising live attenuated vaccine candidate against genotype I/II recombinant ASFV strains, supporting the use of multigene deletion strategies for ASF vaccine development.

**Supplementary Information:**

The online version contains supplementary material available at https://doi.org/10.1186/s13567-026-01827-6.

## Introduction

African swine fever (ASF) is a severely lethal infectious disease affecting both domestic pigs and wild boars. Currently, apart from in Vietnam, no safe, effective, and commercially available vaccine exists for ASF; therefore, disease control in other affected regions continues to rely primarily on culling infected pigs. ASF causes serious damage to the pig industry [1].

The disease was first reported in Kenya in 1921, and its causative factor is African swine fever virus (ASFV). ASFV is a large double-stranded DNA virus that is the only member of the genus *Asfivirus* in the Asfarviridae family [2]. Based on the 3′ terminal sequence of the *B646L* gene encoding the major capsid protein p72, ASFV was classified into 24 different genotypes. The virion is about 260 nm in diameter and has an icosahedral structure, and the viral genome is 170–193 kb and encodes more than 150 open reading frames [3]. Although the gene functions of many viral major proteins have been extensively studied [4], the same gene can still exhibit different virulence across different viral strains. For example, deletion of *I267L* in the ASFV-Gansu (GS) isolate results in loss of virulence in pigs, while this effect is not observed in the ASFV SY18 isolate [5, 6]. This indicates that the gene functions in ASFV are highly intricate. Investigating the functions of the same gene in different viral strains will contribute to the development of live attenuated vaccines against ASF.

Since the first outbreak of ASF in China in 2018 [7], it has been reported in several other Asian countries, such as South Korea [8] and Mongolia [9]. Due to the lack of sufficient safety and effective vaccines, ASF has spread widely and continues to evolve. In 2023, highly lethal genotype I and II recombinant strains were discovered in China. Genomic analysis revealed that over 56% of the genome fragments in these recombinants are derived from genotype II viruses, with the remaining portions originating from genotype I viruses [10]. The JX23-02 strain used in this study belongs to this lineage, sharing >99.9% genome-wide nucleotide identity with the recombinants described by Zhao et al. [10]. Subsequently, similar recombinant viruses have also been reported in Vietnam [11] and Russia [12]. Attenuated live vaccine derived from genotype II cannot protect against the recombinant strains infection [10, 13]. Therefore, it is necessary to develop vaccines targeting recombinant strains.

The development of gene-deleted live attenuated vaccines against ASF has made notable progress. Single gene deletions, including *I73R* [14], *I177L* [15], *MGF505-7R* [16], *A137R* [17], and *I226R* [18], have been shown to protect against genotype II virulent challenge in experimental models. Nevertheless, the safety profiles of such monogenic mutants warrant careful evaluation. Similarly, multigene deletion strains face challenges such as reduced viral replication and suboptimal immunogenicity, which may limit their protective efficacy [19–21]. Recent advances in gene-deleted live attenuated vaccines have been comprehensively reviewed by Niu et al., who summarized the key virulence-related genes and discussed the prospects and challenges for ASFV vaccine development [22]. It is reported that immunization with *A137R* or *I226R* deleted attenuated strains provides full protection against parental genotype II ASFV challenge with a favorable safety profile. Beyond *I226R* and *A137R*, other viral proteins have also been identified as critical modulators of host innate immunity. For instance, the H240R protein suppresses IL-1β production by targeting NF-κB signaling [23], and *I267L* inhibits RNA polymerase III-RIG-I-mediated innate immunity [6]. Mechanistically, *I226R* protein is known to impair antiviral responses, likely through multiple mechanisms including the suppression of NF-κB and IRF3 activation, to counteract innate immune responses [24]. For *A137R*, a virulence-associated gene, inhibits cGAS–STING-mediated IFN-β production by promoting the autophagy-dependent lysosomal degradation of TBK1 [25]. Furthermore, studies indicate that *A137R* may also be implicated in mediating antibody-dependent enhancement (ADE) of infection [26]. Structural analysis reveals that *A137R* forms a dodecahedral cage, potentially contributing to ASFV icosahedral capsid assembly[27]. Of note, sequence analysis of the recombinant strain JX23-02 showed that its *A137R* gene is 100% identical to genotype I strains, while its *I226R* gene is 100% identical to genotype II strains, providing a unique genetic context to evaluate gene deletions in a genotype I/II hybrid background.

Collectively, these findings suggest that, apart from *I177L*, *A137R* and *I226R* represent promising targets for the development of live attenuated ASF vaccines. However, most functional studies on these gene deletions have been conducted in the context of genotype II ASFV, and comparative analysis of their roles in other epidemiologically significant genotypes particularly in emerging genotype I/II recombinant strains is lacking, leaving their genotype-dependent effects on safety and immunogenicity unclear. Moreover, the protective efficacy and safety profile of a recombinant *I226R* or *A137R* deletion in a non-genotype II background remain unexplored. The global spread of ASF continues to threaten the swine industry, with sustained transmission in endemic regions and sporadic incursions into previously free areas [28]. The development of safe and effective vaccines is therefore a global priority. Therefore, to address these gaps, this study was designed to generate and compare isogenic mutants derived from the genotype I/II recombinant strain JX23-02, featuring single deletions of *I226R*, as well as a double *A137R*/*I226R* deletion mutant. We aimed to systematically evaluate the impact of these deletions on viral replication, attenuation, safety, and protective efficacy within this novel recombinant background. This work not only provides critical insights for vaccine design against emerging recombinant strains but also enriches our understanding of the genotype-specific functions of *A137R* and *I226R*.

## Materials and methods

### Cells and viruses

Porcine alveolar macrophages (PAMs) and bone-marrow-derived macrophages (BMDMs) were isolated from the bronchoalveolar lavage fluid and bone marrow aspirates of approximately 2-month-old pigs. The lavage fluids were supplemented with RPMI 1640 medium containing ethylenediaminetetraacetic acid (EDTA) and levofloxacin. After centrifugation, red blood cells were lysed using a red blood cell lysis buffer. The resultant cells were then washed three times with phosphate-buffered saline (PBS) containing levofloxacin and 2% fetal bovine serum (FBS), followed by resuspension in RPMI 1640 medium containing levofloxacin and 10% FBS. For the BMDMs, 10 ng/mL of granulocyte–macrophage colony-stimulating factor (GM-CSF) was added to the resuspension. The cells were cultured for 7 days at 37 °C with 5% CO_2_ in the presence of GM-CSF before use.

ASFV strain JX23-02 (GenBank accession: PP712069) isolated from pig spleen in 2023, is a genotype I and II recombinant variant and preserved in our laboratory [29].

### Plasmid design for homologous recombination

The pMD18-T vector was used as the backbone. An EGFP expression cassette was inserted into the cloning site of pMD18-T. This cassette consisted of: a 1258 bp left homology arm located upstream of the *I226R* open reading frame (ORF), the p72 promoter, the enhanced green fluorescent protein (EGFP) gene, an SV40 poly(A) termination signal, and a 1114 bp right homology arm located downstream of the *I226R* ORF. To preserve the integrity of the *I243L* gene during *I226R* deletion, a 22-bp sequence from the 3′ end of the *I226R* gene was retained. This 22-bp fragment covers the 4-bp overlap between the two ORFs and provides necessary sequence for primer design, while containing no functional domain of *I226R*.

Separately, an mCherry expression cassette was inserted into the cloning site of pMD18-T. This cassette comprised a 1061-bp left homology arm located upstream of the *A137R* ORF, the p72 promoter, the mCherry fluorescent protein gene, an SV40 poly(A) signal, and a 1238-bp right homology arm located downstream of the *A137R* ORF.

### Construction of gene-deletion JX23-02ΔI226R and JX23-02ΔI226RΔA137R

Using the homologous recombination method, the *I226R* gene was replaced with *EGFP* gene to construct the *I226R* gene-deletion recombinant in JX23-02. A total of 2 µg of the recombinant plasmid, which contains the left and right homologous arms and the p72 promoter-operated EGFP gene, were transfected into BMDMs in a 12-well plate. After 6 h, the parental strain was inoculated at a multiplicity of infection (MOI) of 1.0. Upon the emergence of fluorescent cells, a single one was picked under a microscope for isolation, followed by separating the gene-deletion strain from the parental strain using the method of limited dilution. Polymerase chain reaction (PCR) was employed to determine the presence of the parental strain among the obtained strain JX23-02ΔI226R. If the parental strain JX23-02 was present, an 819-bp band would be amplified using the following primers: forward: 5′-CCAATAGGCAACTTTCTTTTG- 3′; reverse: 5′-ACAGGATAACGATGCCCTTA-3′.

Using the same method, a dual-gene deletion virus JX23-02ΔI226RΔA137R was constructed by transfecting a recombinant expression plasmid containing about the *A137R* gene left and right homologous arms and the p72 promoter-operated *mCherry* gene. After transfection, the purified JX23-02ΔI226R was inoculated at an MOI of 1.0. The recombinant virus was purified by picking individual fluorescent cells and employing limited dilution methods. Identification of the recombinant was performed by PCR, which amplified a 389-bp band using the following primers: forward: 5′-CAGTTCTTACCAAACTCGACC-3′; reverse: 5′-CATCTTGCCGATGAGATTTC-3′.

### Viral growth curve assay

To compare the growth curve of JX23-02, JX23-02ΔI226R, and JX23-02ΔI226RΔA137R in porcine alveolar macrophages (PAMs), each virus was inoculated at an MOI of 0.1 in triplicate of PAMs. After incubation for 1.5 h, cells were washed with phosphate-buffered saline (PBS), and samples were collected at 24, 48, 72, 96, and 120 h after infection. Subsequently, PAMs were used to detect the tissue culture infectious dose (TCID_50_) of virus solution at different time points.

The viral titer was detected by the tissue culture infectious dose (TCID_50_) method and the direct immunofluorescence method. PAMs were resuscitated and then seeded into 96-well plates. Twelve hours later, the virus stocks at passages P2–P5 were repeatedly frozen and thawed, followed by tenfold serial dilutions (from 10^1.0^ to 10^8.0^). Subsequently, 100 μL of the diluted virus solution per well was inoculated into the 96-well plates. The cells were continuously cultured for 96 h under the conditions of 37 °C and 5% CO_2_. The cells were then fixed with 4% paraformaldehyde and placed at 4 °C for 20 min. After washing the cells with phosphate-buffered saline (PBS), 0.1% Triton X-100 was added, and the cells were treated for 10 min. The cells were washed again with PBS, and then, the diluted FITC-labeled p30 monoclonal antibody (at a dilution ratio of 1:500) was added. The cells were incubated at 37 °C in the dark for 60 min. After that, the cells were washed three times with PBS with Tween 20 (PBST; 0.05% Tween 20), with each washing lasting for 3 min. The washing solution was discarded, and the number of fluorescent wells was observed and counted under a fluorescence microscope. Finally, the viral titer was calculated according to the Reed–Muench method.

### Animal experiments

To explore the pathogenicity of genotype I/II recombinant strains on pigs, 18 Landrace pigs were randomly divided into four groups. Group A (061, 023, 042, 001, 091), Group B (100, 004, 007, 006, 066), and Group C (275, 276, 277, 278, 279) were the infection groups, with five pigs in each group. Group D (901, 902, 903) was the control group, consisting of three pigs. Pigs in the first three groups were intramuscularly injected with 1 mL of ASFV SY18, 1 mL of HuB20, and 1 mL of JX23-02 in the neck, all at a titer of 100 TCID_50_. Pigs in Group D were injected with 1 mL of PBS as a blank control group. The rectal temperature was measured daily, and their feeding and behaviors were observed. If the infected pigs showed severe clinical symptoms, they were euthanized. The observation lasted for 28 days.

Humane endpoints were predefined as persistent fever (>41 °C for 48 h), complete anorexia, lethargy with inability to stand, or severe dyspnea. Pigs reaching these endpoints were euthanized immediately by intravenous injection of sodium pentobarbital (100 mg/kg).

To assess the effects of deleting the *I226R* gene and both the *I226R* and *A137R* genes on the virulence of the parental JX23-02 strain, 15 Landrace pigs were acquired from local farms for the experiment. The first group of five pigs (810, 812, 813, 817, 818) was intramuscularly inoculated with 10^5.0^ TCID_50_ of JX23-02ΔI226R, while another group of five pigs (081, 082, 083, 084, 085) was immunized with 10^5.0^ TCID_50_ of JX23-02ΔI226RΔA137R. The third group of five pigs (292, 293, 294, 261, 262) served as the challenge control. After inoculation, the clinical symptoms of the animals were monitored on a daily basis. For the surviving pigs during the immunity observation period, a challenge infection was performed via intramuscular injection of the parental strain ASFV JX23-02 at a dose of 100 TCID_50_. Rectal temperature was taken daily, and oral and rectal swabs as well as blood samples were collected for the detection of viral nucleic acid and antibody levels at 0, 3, 7, 10, 14, 17, and 21 days after inoculation and at 0, 3, 7, 10, 14, 17, 21, 24, and 28 days after challenge. A part of the blood samples was preserved in EDTA blood collection tubes for viral load detection, and the other part was used for serum separation to detect antibodies. During the observation period, animals with severe clinical symptom were euthanized, and tissue samples including the heart, liver, spleen, lung, kidney, stomach, lymph nodes (submandibular and inguinal), thymus, tonsils, jejunum, ileum, colon, muscle, and bone marrow were collected. Quantitative polymerase chain reaction (qPCR) was carried out on tissue samples. The animal experiments were conducted in biosafety level 3 laboratories.

### Quantification of the ASFV load

The quantification of p72 gene copies in blood, oral swabs, anal swabs, and tissues was conducted using qPCR protocols recommended by the World Organization for Animal Health (Additional file 1). Viral copy numbers were determined by qPCR using a standard curve based on the p72 gene, as previously described [30]. Tissue samples were cut into approximately 1 cm^3^ pieces and placed into sterile tubes containing steel beads. Subsequently, 1 mL of PBS was added, and the tissues were homogenized at 60 oscillations/s for 30 s using a tissue homogenizer; this cycle was repeated three times with 10-s intervals between cycles. The homogenates were then centrifuged at 12 000 × *g* for 5 min at 4 °C. The supernatants were collected, transferred to new sterile microcentrifuge tubes, and labeled for subsequent use.

Briefly, DNA was extracted from each sample using an animal virus DNA extraction kit. Quantitative PCR (qPCR) was performed using the Premix Ex Taq (Probe qPCR) kit. The primers and probes used are listed in Supplementary Table 1. The copy numbers of ASFV genomic DNA in samples were calculated on the basis of a standard curve established using a standard plasmid containing the ASFV *B646L* gene. The standard curve for the ASFV p72 gene in the quantitative PCR was fitted to the equation *Y* = −3.245*X* + 38.439, where *Y* represents the cycle threshold (Ct) value and *X* represents the copy number of the p72 gene.

**Antibody detection**

The indirect enzyme-linked immunosorbent assay (ELISA) method, developed in our laboratory, was used to detect the level of ASFV p54-specific antibodies in serum [31]. The ELISA plate was coated with p54 antibody and incubated overnight at 4 °C. After blocking, a 50-fold diluted sample serum was added and incubated at 37 °C for 30 min. The plate was washed five times with PBS, then incubated with horseradish peroxidase (HRP)-labeled sheep anti-pig IgG at 37 °C for 30 min. Following the wash, 80 µL of chromogenic solution 3,3′,5,5′-tetramethylbenzidine (TMB) substrate was added to each well and incubated at room temperature in the dark for 10 min; then, the reaction was stopped with 2 M sulfuric acid and read within 3 min. Each serum sample was tested three times, and their absorbance values were measured at 450 nm.

### Statistical analysis

GraphPad Prism 9.5 software was used for graphing and statistical analysis. A *t*-test was employed for significance analysis.

## Results

### The pathogenicity of JX23-02

**Figure 1:**
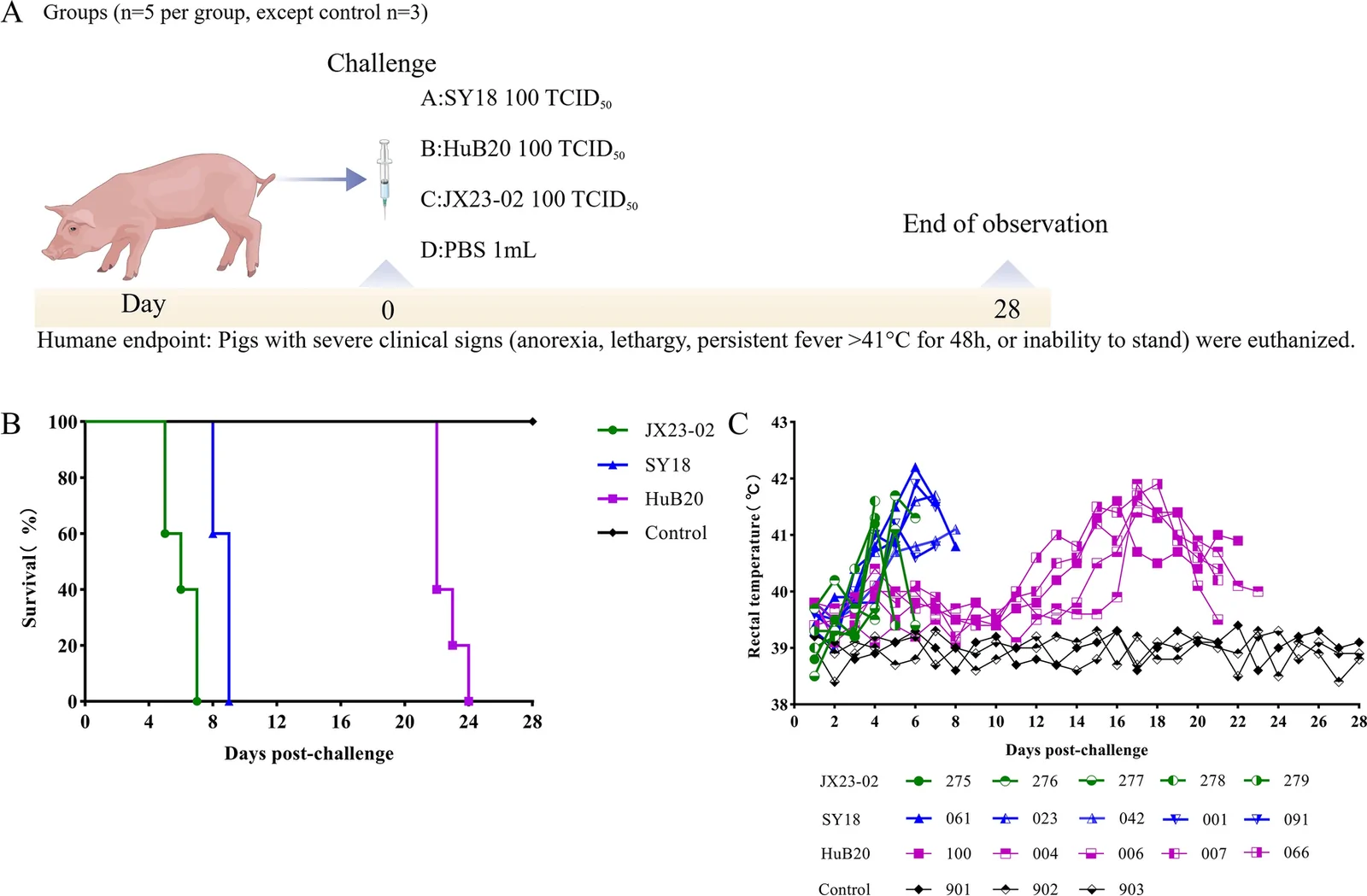
**High pathogenicity of JX23-02 in pigs**. **A** Experimental timeline for pathogenicity evaluation. Pigs were infected intramuscularly with 100 TCID_50_ of the indicated ASFV strains or PBS (control). Clinical signs and rectal temperature were recorded daily. Humane endpoints were applied as described in “Materials and methods”. **B** Survival curves post-challenge with JX23-02, SY18, or HuB20. **C** Body temperature profiles post-challenge with the indicated strains.

Georgia07-like genotype II strain SY18 is a highly lethal strain and HuB20 is naturally attenuated genotype II strain. To determine genotype I/II recombinant strain virulence with genotype II, pigs were infected with the strains of JX23-02, HuB20 [32](GenBank accession:MW521382), and SY18 [7](GenBank accession:MH766894) at a dose of 100 TCID_50_ each, and the rectal temperature was measured daily. Pigs in the control group injected with 1 mL PBS remained clinically normal throughout the experiment (Figure 1A). These three strains demonstrated a 100% mortality rate (Figure 1B). Among them, the pigs infected with the JX23-02 strain developed fever as early as 2 days post-infection (dpi), and the fever lasted for 1–3 days, with the earliest pig reaching the humane endpoint and being euthanized at 5 dpi. The pigs infected with the SY18 strain developed fever at 3 dpi; the fever persisted until around 5 dpi and reached humane endpoints as early as 7 dpi. The pigs infected with the HuB20 strain developed fever at 11 dpi, which lasted for about 10 days, and reached humane endpoints as early as 21 dpi (Figure 1C).

### Construction and growth characteristics of JX23-02ΔI226R and JX23-02ΔI226RΔA137R deletion strains

**Figure 2:**
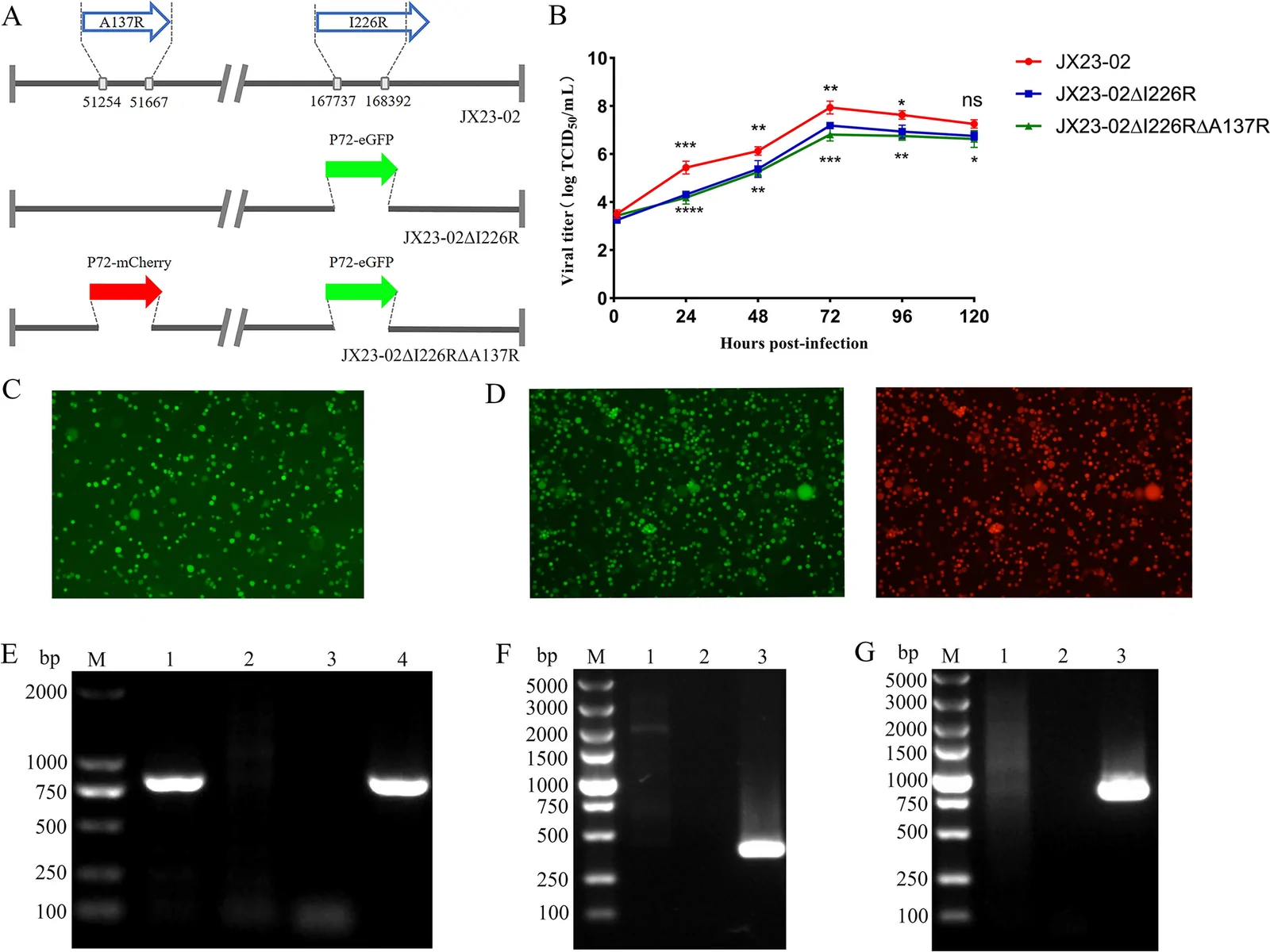
**The construction of gene-deleted ASFV JX23-02ΔI226R and JX23-02ΔI226RΔA137R**. **A** Schematic representation of JX23-02ΔI226R and JX23-02ΔI226RΔA137R construction. **B** In vitro replication kinetics of gene-deleted ASFV JX23-02ΔI226R and JX23-02ΔI226RΔA137R compared with ASFV JX23-02. Data are shown as mean ± standard deviation (SD) (*n* = 3 independent experiments). “ns” indicates no significant difference, “*” represents a significant difference (*P *< 0.05), “**” indicates an extremely significant difference (*P *< 0.01), and “***” represents an extremely significant difference (*P *< 0.001). **C** The ASFV JX23-02ΔI226R-infected BMDMs are indicated by EGFP reporter fluorescence. **D** The ASFV JX23-02ΔI226RΔA137R-infected BMDMs are indicated by EGFP and mCherry reporter fluorescence. **E** The ASFV JX23-02ΔI226R genome is amplified using specific primers targeting *I226R*. The ASFV JX23-02 genome is used as a positive control. Lane 1–2: *I226R* gene amplification from purified JX23-02ΔI226R samples; Lane 3: negative control; and Lane 4: *I226R* gene in ASFV JX23-02. **F** The ASFV JX23-02ΔI226RΔA137R genome is amplified using specific primers targeting *A137R*. Lane 1: *A137R* gene amplification from purified JX23-02ΔI226RΔA137R samples; Lane 2: negative control; and Lane 3: *A137R* gene in ASFV JX23-02. **G** The ASFV JX23-02ΔI226RΔA137R genome is amplified using specific primers targeting *I226R*. Lane 1: *I226R* gene amplification from purified JX23-02ΔI226RΔA137R samples; Lane 2: negative control; and Lane 3: *I226R* gene in ASFV JX23-02.

The construction strategy of the ASFV gene-deleted strain is illustrated in Figure 2A, where the *I226R* gene was replaced with the p72-EGFP cassette via homologous recombination. Since the terminal ends of the *I226R* and *I243L* open reading frames (ORFs) share four base pairs, when deleting the *I226R* ORF, it is necessary to avoid disrupting the *I243L* ORF. Therefore, 673 base pairs of the *I226R* were deleted to protect the integrity of the *I243L* ORF. After transfection and infection of BMDMs, the fluorescent cell lesions were collected. After multiple rounds of limiting dilution, virus expressing green fluorescence was purified and named JX23-02ΔI226R (Figure 2C). Routine polymerase chain reaction (PCR) was performed on the purified virus using differential primers, and no band of 819 base pairs in size was generated, indicating that the purification was successful (Figure 2E). The *A137R* gene was completely deleted on the basis of the JX23-02ΔI226R strain, and the *A137R* gene locus was replaced with the p72-mCherry cassette. After purification, a recombinant virus emitting both red and green fluorescence was obtained and designated as JX23-02ΔI226RΔA137R (Figure 2D). Through PCR identification, the purified JX23-02ΔI226RΔA137R strain was obtained (Figure 2F, G).

To elucidate the effects of I226R and A137R deletion on ASFV JX23-02 replication, we evaluated the replication kinetics of JX23-02ΔI226R and ASFV JX23-02ΔI226RΔA137R in BMDMs. JX23-02ΔI226R or ASFV JX23-02ΔI226RΔA137R was infected at an MOI of 0.01, and the virus yields were detected at 0, 24, 48, 72, 96, and 120 h post-infection (hpi), respectively. The results showed that, compared with the parental strain, the viral titers of the recombinant strains JX23-02ΔI226R and JX23-02ΔI226RΔA137R were significantly reduced at 24 hpi, 48 hpi, and 72 hpi (*P *< 0.01). At 96 hpi and 120 hpi, the viral titers of the parental strain were significantly higher (*P *< 0.05) than those of the double gene-deleted strain JX23-02ΔI226RΔA137R, while there was no significant difference in the titers between the single gene-deleted strain JX23-02ΔI226R and the parental strain at 120 hpi (*P *> 0.05). In addition, the titers of the double gene-deleted strain JX23-02ΔI226RΔA137R were slightly lower than those of the single gene-deleted strain JX23-02ΔI226R at 72 hpi and 96 hpi, but there was no statistically significant difference between them (*P *> 0.05). Overall, the deletion of the *I226R* and *A137R* genes had a stage-specific effect on the in vitro replication ability of the ASFV JX23-02 strain, especially during the middle stage of infection (24–72 hpi). The specific results are shown in Figure 2B.

### JX23-02ΔI226R exhibits residual virulence but provides protection in surviving pigs

**Figure 3:**
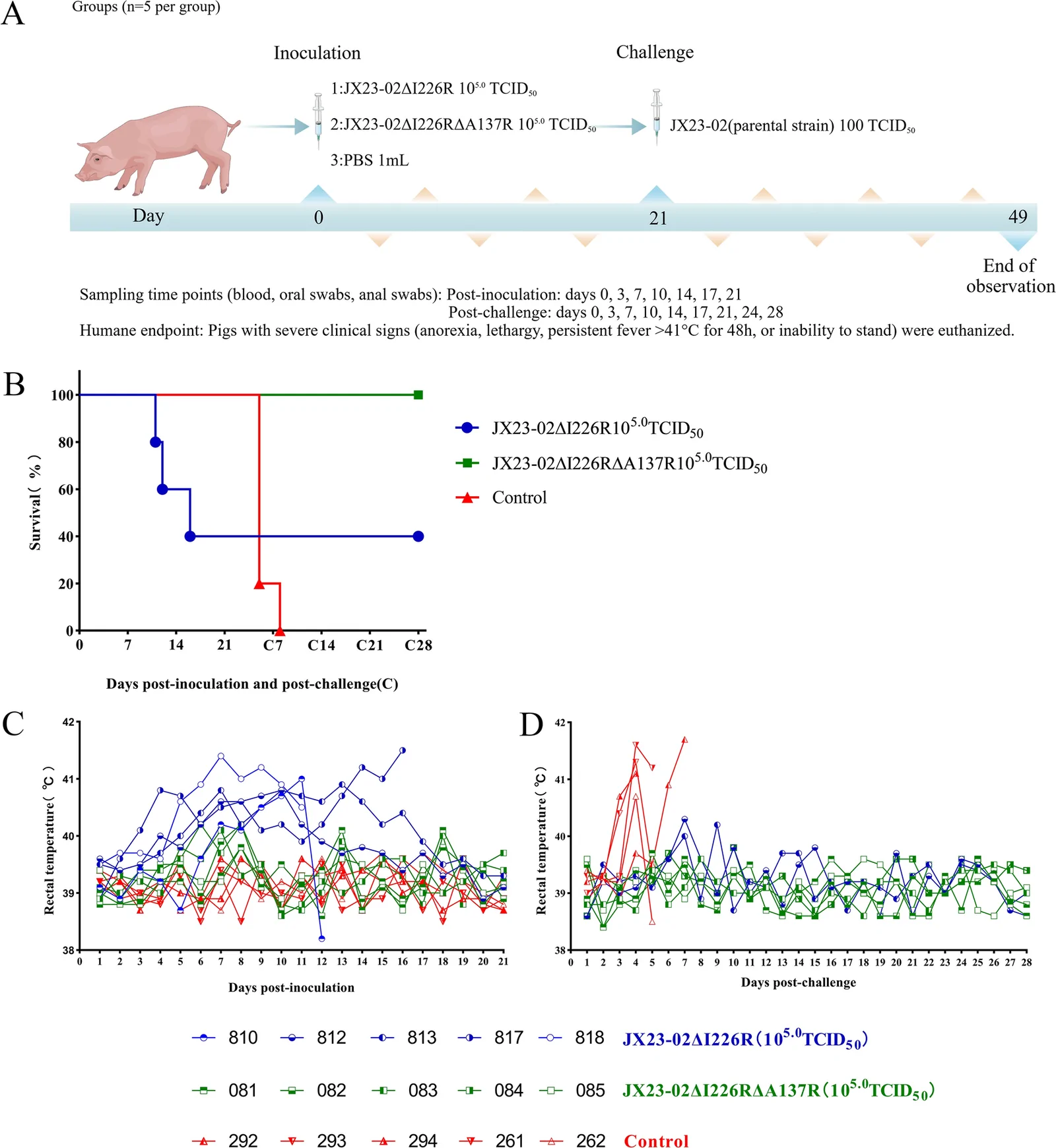
**Survival and body temperature responses to inoculation and challenge**. **A** Experimental design timeline. Pigs (*n* = 5 per group) were immunized with 10^5.0^ TCID_50_ of JX23-02ΔI226R, JX23-02ΔI226RΔA137R, or PBS. At 21 days post-inoculation, all pigs were challenged with 100 TCID_50_ of parental JX23-02. Sampling was performed at the indicated time points. **B** Survival curves post-inoculation and post-challenge. **C** Body temperature profiles post-inoculation. **D** Body temperature profiles post-challenge.

To evaluate the safety and protective efficacy of the single gene-deleted mutant JX23-02ΔI226R, five pigs were immunized intramuscularly with a dose of 10^5.0^ TCID_50_ of JX23-02ΔI226R. Three pigs (810, 817, 818) reached humane endpoints and were euthanized at 11, 12, and 16 dpi, respectively. The deceased pigs exhibited typical clinical signs of ASF, including fever, depression, lethargy, and anorexia, with peak body temperatures reaching 41.5 °C [33]. The remaining two pigs showed normal feeding behavior and mobility within the 21-day observation period, resulting in a survival rate of 40% (Figure 3B). The pigs started to develop fever on the third day after immunization, with the fever reaching its peak from 7 to 10 dpi and lasting for 5–13 days. Two pigs exhibited pyrexia, with individual peak temperatures reaching 40.9 °C and 40.8 °C (Figure 3C). The febrile response was accompanied by anorexia and reduced mobility, and both animals recovered fully within 3–4 days after symptom onset.

21 days after immunization, the pigs were challenged intramuscularly with a dose of 100 TCID_50_ JX23-02. The body temperatures of the two surviving pigs immunized with JX23-02ΔI226R increased transiently on 7–9 days post-challenge (dpc) and then returned to normal (Figure 3D). They survived with normal physiological performance within the 28-day observation period. In the control group, the body temperatures of the pigs increased sharply on the 3rd day after challenge. Pigs reached humane endpoints and were euthanized between 5 and 8 dpc (Figure 3B).

In summary, immunization with JX23-02ΔI226R resulted in residual virulence causing 60% mortality, but surviving pigs exhibited protective efficacy against lethal challenge with the parental virus.

### JX23-02ΔI226RΔA137R is attenuated and provides protective efficacy against the parental virus strain, with 100% survival and no clinical signs

To evaluate the safety and protective efficacy of the double gene-deleted mutant JX23-02ΔI226RΔA137R, five pigs were immunized intramuscularly with a dose of 10^5.0^ TCID_50_ of JX23-02ΔI226RΔA137R. All pigs survived during the immunization observation period and also after the challenge with the parental virus strain. The pigs started to have a slight fever on the fifth day after immunization, which lasted for 1–3 days. There was some fluctuation in body temperature during the observation period, but the maximum body temperature did not exceed 40.1 °C. A total of 21 days after immunization, the five pigs were challenged with 100 TCID_50_ of the parental virus strain JX23-02. All pigs survived (Figure 3B). Throughout the experimental period, their rectal temperatures ranged from 38.4 °C to 40.2 °C, with only transient mild elevations (peak 40.2 °C) observed on days 6–8 post-immunization (Figure 3C and D). No pig exhibited sustained fever or other clinical signs of ASF.

Overall, JX23-02ΔI226RΔA137R was fully attenuated, and all immunized pigs survived challenge with the parental genotype I/II recombinant ASFV strain without developing clinical signs.

### Occasional shedding occurs through the oral or anal route after JX23-02ΔI226R and JX23-02ΔI226RΔA137R inoculation and ASFV JX23-02 challenge

**Figure 4:**
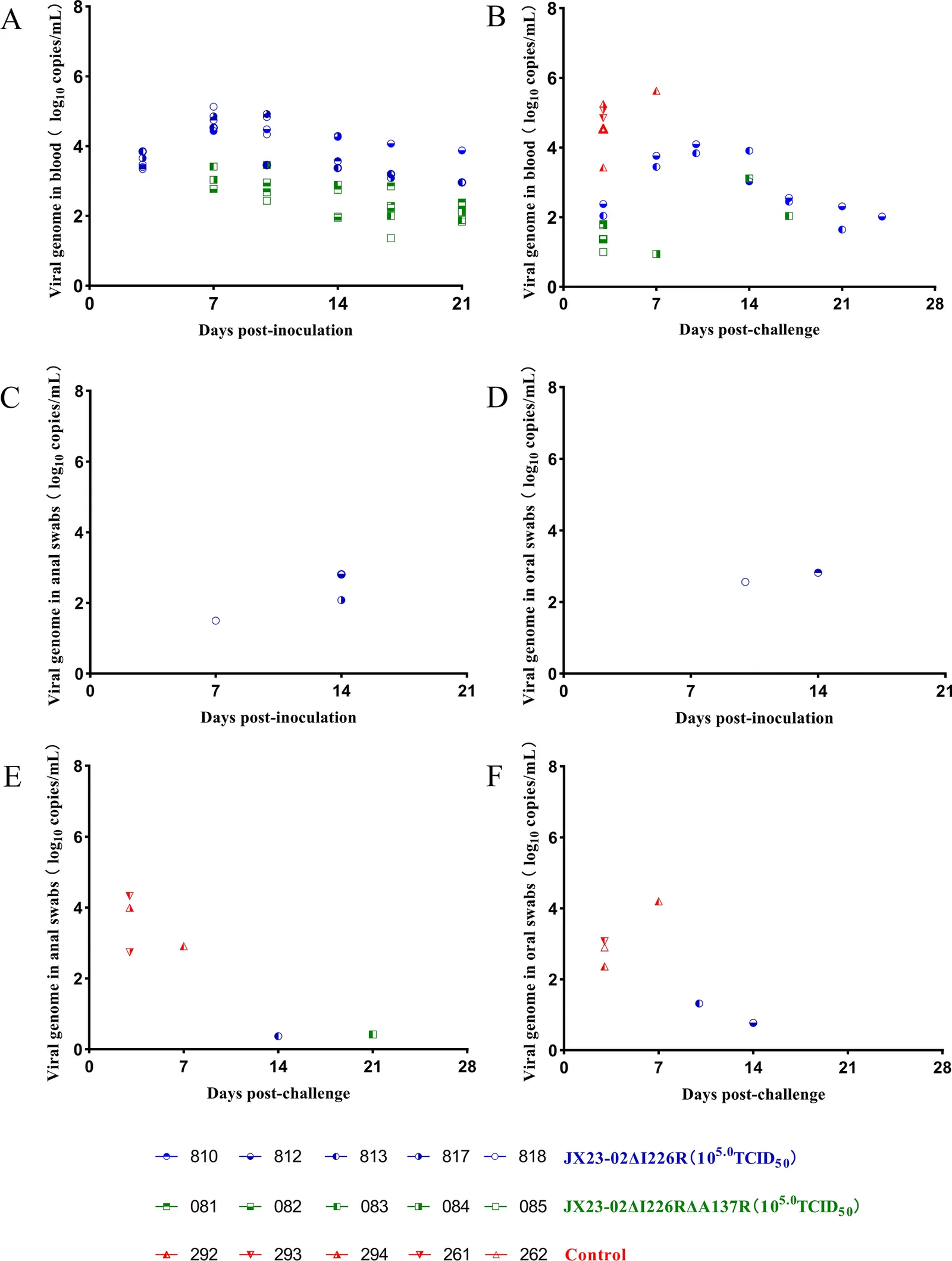
**Viral genomic DNA detection in blood, oral swabs, and anal swabs from the three groups of pigs**. (**A**, **B**) Viral genome copies in blood post-inoculation and post-challenge, respectively. (**C**, **D**) Viral genome copies in anal and oral swabs post-inoculation. (**E**, **F**) Viral genome copies in anal and oral swabs post-challenge.

During the observation period after immunization and challenge, oral and anal swabs were collected from pigs, DNA was extracted, and viral load was detected by qPCR. The detection results of oral and anal swabs after immunization are shown in Figure 4. In the pigs immunized with JX23-02ΔI226R, the oral (Figure 4D) and anal (Figure 4C) swabs showed positive results in four pigs during the early stage (within 7–14 days) after vaccination, and viremia was detected in all these pigs (Figure 4A). In contrast, in the pigs immunized with JX23-02ΔI226RΔA137R, all swab samples tested negative (Figure 4C, D).

The amplification results of the p72 gene on the oral and anal swabs after challenge infection showed that, in the control group pigs, viral shedding was detected starting from 3 days after challenge, and the copy number of viral DNA was relatively high. In the oral and anal swabs of the JX23-02ΔI226R immunized group, viral shedding was detected in 1–2 pigs (Figure 4E, F). In the JX23-02ΔI226RΔA137R immunized group, viral DNA was only detected in the anal swab of one pig, showing a weakly positive result (Figure 4E). This suggests that JX23-02ΔI226RΔA137R may have an improved safety profile compared with JX23-02ΔI226R.

### JX23-02ΔI226RΔA137R induces lower viremia and tissue viral load compared with JX23-02ΔI226R

**Figure 5:**
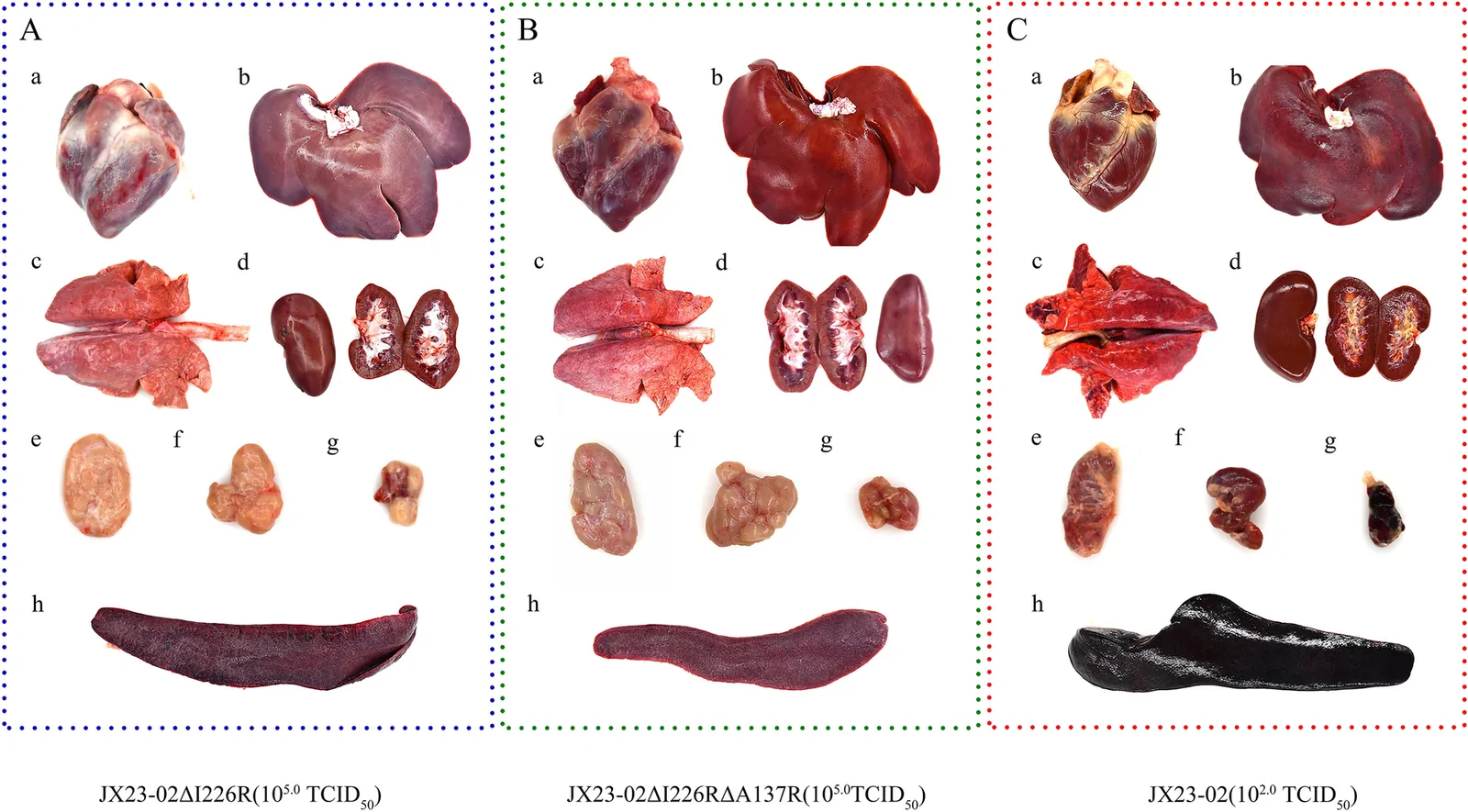
**Anatomical characteristics of porcine tissues and organs**: heart (**a**), liver (**b**), lung (**c**), kidney (**d**), inguinal lymph node (**e**), submandibular lymph node (**f**), thymus (**g**), and spleen (**h**).

We performed qPCR on the whole blood of pigs collected after immunization and challenge to detect the viral genome content. Autopsies were carried out on each pig that reached humane endpoints. All organs of these animals in the control group and the JX23-02ΔI226R immunization group showed typical gross pathological changes of African swine fever, such as splenomegaly and visceral hemorrhage (Figure 5A, C). Aside from a few petechial hemorrhages observed in the lungs, no obvious pathological changes were observed in the pigs of the JX23-02ΔI226RΔA137R immunization group. (Figure 5B). Organ tissue samples were collected for quantitative detection of the viral genome.

In the blood samples after immunization, viral DNA was detected at high levels in the blood of control group pigs after challenge, and the viral DNA copy number in various tissues was also high. The viral content in the blood of pigs immunized with JX23-02ΔI226R was significantly higher than that in the JX23-02ΔI226RΔA137R group. The viral genome in the blood was undetectable (negative) by qPCR 28 days after challenge. In the JX23-02ΔI226R group, the viral load in the tissues of pigs that were euthanized after immunization was high, and qPCR was positive in all types of tissue samples. In the surviving pigs, the virus was detected in the colon, jejunum, and bone marrow, indicating that the virus in the tissues and organs could not be completely cleared.

In the JX23-02ΔI226RΔA137R group, viremia disappeared 21 days after challenge, and the blood qPCR test was negative. In the detection of the viral genome in tissue samples, the kidney, inguinal lymph node, thymus, and stomach tissues tested negative in all pigs. Viral genome was detected in other tissues, but only in a limited number of samples. Specifically, spleen and bone marrow samples were positive in two pigs, with viral loads not exceeding 3.0 log_10_ copies/mL in the spleen and 2.0 log_10_ copies/mL in the bone marrow. The highest viral load among all tissues was observed in the submaxillary lymph nodes (3.11 log_10_ copies/mL).

### JX23-02ΔI226R and JX23-02ΔI226RΔA137R induced strong immune response

**Figure 6:**
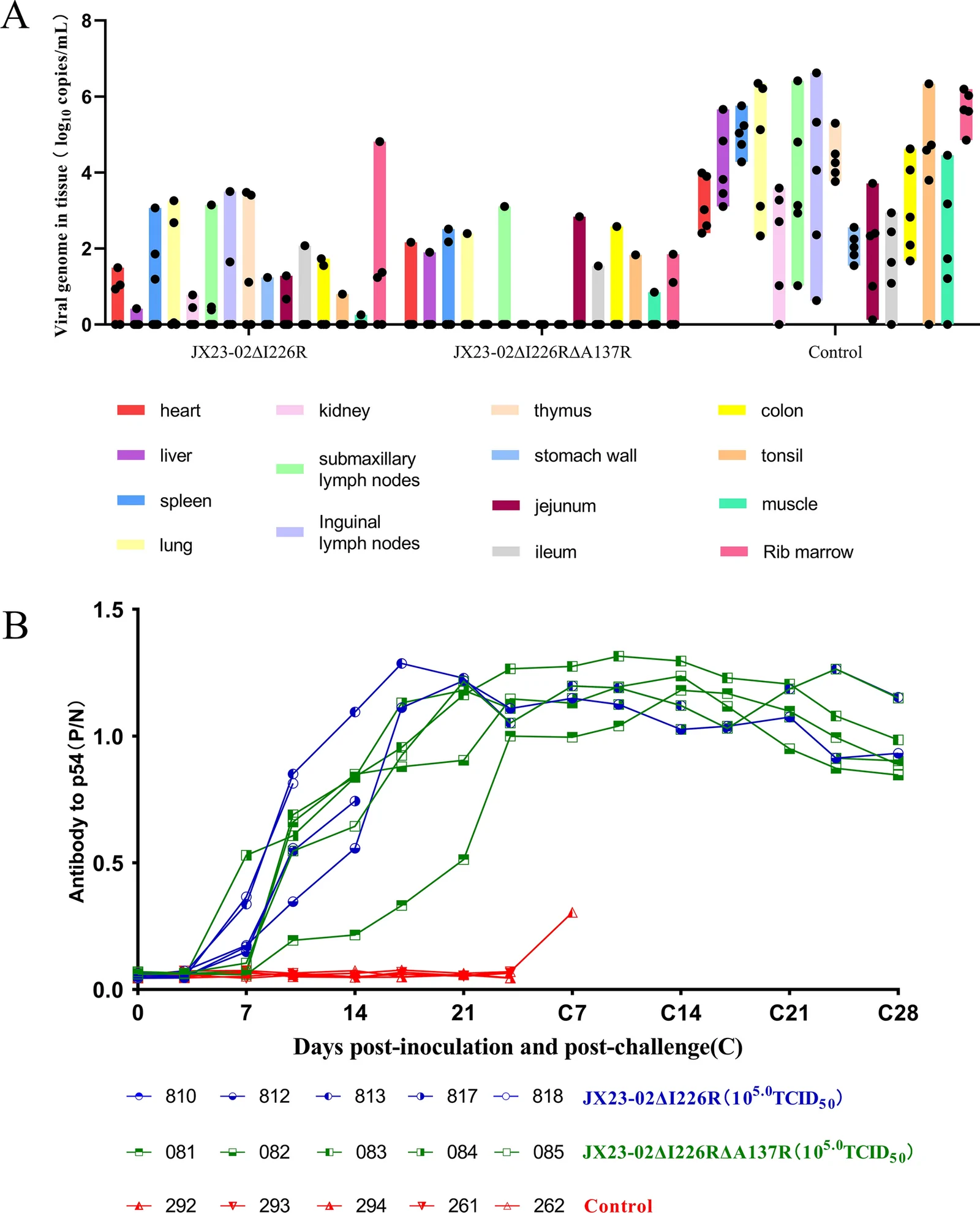
**ASFV genomic copy numbers in tissues and serum antibody detection results**. **A** ASFV genomic copy numbers in tissues collected from pigs at necropsy. **B** Antibody to p54 response curves of different groups of pigs after inoculation and challenge.

The kinetics of p54-specific antibody production were consistent with those typically observed for attenuated ASFV vaccines. As illustrated in Figure 6B, antibody levels were detectable in all experimental groups on day 7 post-vaccination, peaking approximately between days 17 and 21 post-vaccination. Following challenge, the p54 antibody levels in the experimental groups slightly increased and then declined moderately over time, but remained at a high positive level throughout the observation period. In contrast, p54 antibodies were only detected at a low level in the control group after challenge.

## Discussion

In the present study, we successfully constructed a single gene-deleted strain (JX23-02ΔI226R) and a double gene-deleted strain (JX23-02ΔI226RΔA137R) derived from the genotype I/II recombinant ASFV strain JX23-02. The emergence of genotype I/II recombinant ASFV strains poses new challenges for vaccine development, as existing genotype II-based vaccines have shown limited protection against these variants [10]. Our successful attenuation of the recombinant strain JX23-02 through gene deletion demonstrates that targeted modification of circulating field strains is a feasible strategy to address this challenge. Subsequently, we systematically evaluated their immunogenicity, protective efficacy, and safety as potential vaccine candidates. A key finding of this study is that the deletion of *I226R* and *A137R* genes significantly impaired the in vitro replication capacity of ASFV, particularly during the middle stage of infection (24–72 h), as evidenced by a marked reduction in viral titer. This observation provides critical theoretical insights into the development of attenuated ASFV vaccines, highlighting the potential of *I226R* and *A137R* as key virulence-related targets for viral attenuation.

Animal experiments revealed distinct levels of immune protection among the constructed strains. Specifically, JX23-02ΔI226R retained residual virulence, which led to the death of some immunized pigs; however, it still induced effective protective immune responses against the parental strain, indicating its potential as a vaccine candidate with room for further optimization. In contrast, JX23-02ΔI226RΔA137R exhibited superior safety profiles: all immunized pigs survived the challenge without manifesting obvious clinical symptoms. This result demonstrates that the additional deletion of the *A137R* gene further attenuated viral virulence without compromising immunogenicity, confirming the synergistic effect of dual gene deletion on ASFV attenuation. Furthermore, qPCR analysis showed that pigs immunized with JX23-02ΔI226RΔA137R had significantly shorter duration of viremia and lower viral load compared with those in the JX23-02ΔI226R group. These data further validate the advantages of JX23-02ΔI226RΔA137R in suppressing viral replication and reducing viremia persistence, thereby providing robust support for its potential as a promising vaccine candidate. The enhanced safety profile of JX23-02ΔI226RΔA137R may be attributed to the cumulative effect of deleting two immunomodulatory genes. *I226R* suppresses NF-κB and IRF3 activation [24], while A137R inhibits cGAS–STING-mediated IFN-β production by promoting autophagy-dependent degradation of TBK1 [25]. Simultaneous deletion of both genes likely leads to more robust innate immune responses against the virus, thereby limiting its replication and dissemination in vivo, which explains the reduced viremia and lower tissue viral load observed in this group. Collectively, on the basis of reduced viremia, lower tissue viral load, and absence of mortality, JX23-02ΔI226RΔA137R outperformed JX23-02ΔI226R in terms of safety, immunogenicity, and viral replication control, offering valuable experimental basis and theoretical support for the development of ASFV vaccines.

Our findings exhibit both similarities and discrepancies with previous reports on ASFV gene-deleted vaccines. Zhang et al.[18] demonstrated that the SY18ΔI226R strain did not induce obvious clinical signs after vaccination, and all vaccinated pigs survived lethal challenge without clinical signs. While JX23-02ΔI226R in the present study also provided partial protective efficacy, its higher residual virulence resulted in partial mortality among immunized animals. This discrepancy is likely attributed to the inherent genetic background differences and distinct pathogenic mechanisms between the parental strains (SY18 and JX23-02), emphasizing the importance of tailoring attenuation strategies to specific ASFV genotypes or recombinant strains. However, Zhao et al. reported that the genotype II attenuated live vaccine HLJ/18-7GD failed to protect against genotype I/II recombinant ASFV strains, suggesting that genomic recombination of ASFV may lead to the emergence of novel epitopes or immune escape mechanisms, thereby compromising the protective efficacy of existing vaccines [10]. The successful development of JX23-02ΔI226RΔA137R in this study addresses this challenge by proposing a multigene deletion strategy, which achieves enhanced attenuation while preserving critical immunogenic determinants. Recently, Peng et al. demonstrated that deletion of *CD2v* and *A137R* from another genotype II strain also resulted in substantial attenuation, with all vaccinated pigs surviving without clinical signs or fever (> 40.5 °C) [34]. Additionally, Gladue et al. showed that the single deletion of A137R attenuates a genotype II strain, but only at a low dose (10^2.0^ HAD_50_) [17]. In contrast, JX23-02ΔI226RΔA137R achieved full attenuation at a 1000-fold higher dose (10^5.0^ TCID_50_), indicating that simultaneous deletion of I226R and A137R provides enhanced safety. While their double-deletion strategy targeted different genes (*CD2v* and *A137R*), our results with JX23-02ΔI226RΔA137R similarly confirm that *A137R* is a key target for attenuation across different genetic backgrounds, and suggest that multiple gene combinations can achieve optimal safety and efficacy.

Several limitations should be acknowledged. First, the potential for reversion to virulence and the duration of protective immunity remain to be evaluated in future studies. Second, although our qPCR detected viral DNA after challenge, it does not distinguish between vaccine and challenge virus. Third, co-infection with field strains could theoretically generate recombinants, and cross-protection against genotype II strains requires further investigation. Despite these limitations, JX23-02ΔI226RΔA137R exhibited favorable safety and protective efficacy profiles, supporting its further development. The complete deletion of *A137R* in JX23-02ΔI226RΔA137R provides a potential Differentiating Infected from Vaccinated Animals (DIVA) marker. Antibodies against *A137R* are present in wild-type ASFV-infected pigs but absent in vaccinated animals, allowing serological differentiation between infection and vaccination. This feature is valuable for future field application and disease surveillance.

In conclusion, we successfully constructed two gene-deleted strains (JX23-02ΔI226R and JX23-02ΔI226RΔA137R) based on the genotype I/II recombinant ASFV strain JX23-02. Deletion of *I226R* and *A137R* significantly attenuated viral replication capacity. Among the candidates, JX23-02ΔI226RΔA137R exhibited excellent performance in safety, immunogenicity, and protective efficacy, whereas JX23-02ΔI226R induced partial protective immunity but retained undesirable residual virulence. These results highlight the feasibility of multigene deletion as a promising approach for the development of safe and effective ASFV vaccines, especially against emerging recombinant strains.

## Supplementary Information


 **Additional file 1 Primers and probes used for qPCR detection of ASFV genes.** A table listing the forward and reverse primer sequences and the probe sequence for the ASFV p72 gene qPCR assay.**Additional file 2 Whole-genome sequence comparison of JX23-02ΔI226R with the parental JX23-02 strain.** A table detailing the genomic loci, nucleotide changes, and mutational regions identified in the single gene-deleted recombinant virus.**Additional file 3 Whole-genome sequence comparison of JX23-02ΔI226RΔA137R with the parental JX23-02 strain.** A table detailing the genomic loci, nucleotide changes, and mutational regions identified in the double gene-deleted recombinant virus.

## Data Availability

The genome sequences of ASFV strains JX23-02, JX23-02ΔI226R, and JX23-02ΔI226RΔA137R have been deposited in GenBank under accession numbers PP712069, PZ498702, and PZ498701, respectively. Sequence comparisons are provided in Supplementary Tables 2 and 3.
