# Supplementary material for: Evaluation of An I226R and A137R deletion mutant from a genotype I/II recombinant African swine fever virus strain as a live attenuated vaccine in pigs
Source: Vet Res. 2026 Aug 28;57:159. doi: 10.1186/s13567-026-01827-6 (PMC13525649; doi:10.1186/s13567-026-01827-6)
Supplement: Supplementary file 1 [file 13567_2026_1827_MOESM1_ESM.docx]

Additional Table1. Primers and probes used for qPCR detection of ASFV genes.A table listing the forward and reverse primer sequences and the probe sequence for the ASFV p72 gene qPCR assay.

| Primer/Probe | Sequence (5'→3') | Purpose |
| --- | --- | --- |
| qp72-F | CTGCTCATGGTATCAATCTTATCGA | p72 gene detection |
| qp72-R | GATACCACAAGATCAGCCGT | p72 gene detection |
| Probe-p72 | CCACGGGAGGAATACCAACCCAGTG | p72 gene detection |
