# Supplementary material for: Evaluation of An I226R and A137R deletion mutant from a genotype I/II recombinant African swine fever virus strain as a live attenuated vaccine in pigs
Source: Vet Res. 2026 Aug 28;57:159. doi: 10.1186/s13567-026-01827-6 (PMC13525649; doi:10.1186/s13567-026-01827-6)
Supplement: Supplementary file 2 [file 13567_2026_1827_MOESM2_ESM.docx]

Additional Table 2. Whole-genome sequence comparison of JX23-02ΔI226R with the parental JX23-02 strain. A table detailing the genomic loci, nucleotide changes, and mutational regions identified in the single gene-deleted recombinant virus.

| Genomic Loci | JX23-02  (Reference genome) | JX23-02ΔI226R  (Modified genome) | | Mutational Region |
| --- | --- | --- | --- | --- |
| 5 | C | | G | Left Variable Region |
| 11 | T | | A | Left Variable Region |
| 536 | A | | T | Left Variable Region |
| 566 | A | | - | Left Variable Region |
| 14762 | - | | G | Left Variable Region |
| 186797 | T | | C | Right Variable Region |
| 186799 | T | | G | Right Variable Region |

All identified mutations are located in non-coding regions (left or right variable regions) and do not affect any open reading frames.
