# Supplementary material for: Evaluation of An I226R and A137R deletion mutant from a genotype I/II recombinant African swine fever virus strain as a live attenuated vaccine in pigs
Source: Vet Res. 2026 Aug 28;57:159. doi: 10.1186/s13567-026-01827-6 (PMC13525649; doi:10.1186/s13567-026-01827-6)
Supplement: Supplementary file 3 [file 13567_2026_1827_MOESM3_ESM.docx]

Additional Table 3. Whole-genome sequence comparison of JX23-02ΔI226RΔA137R with the parental JX23-02 strain.A table detailing the genomic loci, nucleotide changes, and mutational regions identified in the double gene-deleted recombinant virus.

| Genomic Loci | JX23-02  (Reference genome) | JX23-02ΔI226RΔA137R  (Modified genome) | | Mutational Region |
| --- | --- | --- | --- | --- |
| 11 | T | | A | Left Variable Region |
| 64 | A | | G | Left Variable Region |
| 79 | A | | T | Left Variable Region |
| 530 | T | | A | Left Variable Region |
| 16851 | G | | T | Left Variable Region |
| 186745 | T | | A | Right Variable Region |

All identified mutations are located in non-coding regions (left or right variable regions) and do not affect any open reading frames.
